# PREVALENCE, Characteristics, and Awareness of Chronic Kidney Disease in Croatia: The EH-UH 2 Study

**DOI:** 10.3390/jcm13226827

**Published:** 2024-11-13

**Authors:** Ana Jelaković, Danilo Radunović, Josipa Josipović, Tajana Željković Vrkić, Lana Gellineo, Marija Domislović, Vladimir Prelević, Marijana Živko, Mirjana Fuček, Mihaela Marinović Glavić, Nikolina Bašić-Jukić, Ivan Pećin, Marija Bubaš, Krunoslav Capak, Bojan Jelaković

**Affiliations:** 1 Department for Nephrology, Hypertension, Dialysis and Transplantation, University Hospital Center Zagreb, 10000 Zagreb, Croatia; anajelakovic9@gmail.com (A.J.); danilo.radunovic@kccg.me (D.R.); tajana.zeljkovic.vrkic@kbc-zagreb.hr (T.Ž.V.); lana.gellineo@gmail.com (L.G.); marija.domislovic@kbc-zagreb.hr (M.D.); vladimir.scopheuope@gmail.com (V.P.); marijana.zivko3@gmail.com (M.Ž.); nbasic@kbc-zagreb.hr (N.B.-J.); 2 Department of Social Medicine and Epidemiology, Faculty of Medicine, University of Rijeka, 51000 Rijeka, Croatia; mihaela.marinovic@uniri.hr; 3 Clinic for Nephrology, Clinical Centre of Montenegro, 81000 Podgorica, Montenegro; 4 Department of Nephrology and Hypertension, Sestre Milosrdnice University Hospital Centre, 10000 Zagreb, Croatia; 5 School of Medicine, Catholic University of Croatia, 10000 Zagreb, Croatia; 6 Department of Laboratory Diagnostics, University Hospital Center Zagreb, 10000 Zagreb, Croatia; mirjana.fucek@kbc-zagreb.hr; 7 School of Medicine, University of Zagreb, 10000 Zagreb, Croatia; ivanpecin@yahoo.com; 8 Department of Metabolism, University Hospital Center Zagreb, 10000 Zagreb, Croatia; 9 Croatian Institute of Public Health, 10000 Zagreb, Croatia; marija.bubas@miz.hr (M.B.); kcapak@hzjz.hr (K.C.); 10 Ministry of Health of the Republic of Croatia, 10000 Zagreb, Croatia

**Keywords:** albuminuria, awareness, chronic kidney disease, cardiovascular risk, eGFR (CKD-EPI) formula, glomerular filtration rate, national population survey

## Abstract

**Background**. National surveys have reported variable prevalence of chronic kidney disease (CKD), due to differences in the characteristics of the population, study design, equations used for the estimated glomerular filtration rate (eGFR), and definitions. The EH-UH 2 survey is the first study evaluating CKD prevalence, characteristics, and awareness in Croatia. **Methods**. This was a cross-sectional nationwide observational study designed to assess the prevalence of CKD and cardio–kidney–metabolic risk factors in Croatia, which included 1765 randomly selected subjects. We estimated the prevalence of CKD by means of the albumin-to-creatinine ratio (ACR) and eGFR (CKD-EPI equation). Comorbidities and anthropometric and social factors related to the prevalence of CKD were analyzed, and the CV risk profile was evaluated. **Results**. The weighted prevalence of CKD (any stage), CKD stage ≥G3A A2, and CKD defined only as an eGFR <60 mL/min/1.73 m^2^ were estimated at 17.1%, 9.8%, and 7.9%, respectively. The prevalence was higher in men than in women (11.8% vs. 7.9%; *p* < 0.001). The weighted prevalence of an ACR >30 mg/g was 15.1%. Older age, male gender, diabetes, ePWV, and uric acid were independently associated with CKD prevalence. The awareness of CKD was 9.5%. Persons unaware of CKD were older with lower income, less education, more frequent diabetes, hypertension (less frequently controlled), and milder renal impairment. **Conclusions**. In Croatia, the estimated prevalence of CKD is high, being presented more frequently in men than in women. CKD patients have an unfavorable CV risk profile. The awareness of CKD is very low, reflecting poor health literacy in the general population but also in health-care workers.

## 1. Introduction

To assess the burden of kidney dysfunction, the prevalence of chronic kidney disease (CKD) in 2017 was estimated as 9.1% in the world’s population [1]. In that analysis, the age-standardized prevalence of CKD in Croatia was estimated to be 8.8%. In other analyses that included 33 population-based representative studies from around the world, the age-standardized global prevalence of CKD stages 1–5 in subjects older than 20 years was 10.4% among men and 11.8% among women [2]. The current total number of individuals affected by CKD stages 1–5 worldwide is estimated to be 843.6 million, which is more people than those with diabetes, osteoarthritis, chronic obstructive pulmonary disease (COPD), asthma, or depressive disorders [3,4]. Disturbingly, the burden of kidney disease is rising worldwide. According to the Global Burden of Disease (GBD) Study, the global prevalence of CKD increased by 33% between 1990 and 2017 [1]. Death and disability due to CKD have increased as well. Globally, the CKD burden is mostly driven by population growth, aging, diabetes, and hypertension [5]. CKD disproportionately affects vulnerable and marginalized populations, and most of the burden is concentrated in the three lowest quintiles of the socio-demographic index.

CKD has been recognized as an independent risk factor for cardiovascular disease (CVD) and a risk multiplier in patients with hypertension and diabetes [6,7,8,9]. In 2017, impaired kidney function resulted in 61.3 million DALYs, of which 58.4% were directly attributable to CKD, whereas 41.6% were CVD DALYs [1]. Of the CVD DALYs attributable to kidney dysfunction, 58.8% came from ischemic heart disease and 40.2% from stroke [1]. In the 2021 European Society of Cardiology (ESC) guideline on CVD prevention, individuals with moderate and severe CKD are regarded as being at high and very high risk of CVD, respectively [10]. To personalize CVD preventive therapies, the ESC developed and validated a new approach that allowed the inclusion of information on the two CKD measures, the estimated glomerular filtration rate (eGFR) and albuminuria, into existing prediction models. This approach, the CKD add-on, has significantly improved CVD risk prediction beyond the Pooled Cohort Equation (PCE) and SCORE [11,12,13]. Recently, the American Heart Association launched a new holistic paradigm on cardio–kidney–metabolic health and a new predictive equation (PREVENT), also emphasizing the importance of kidney dysfunction for global risk [14].

CKD is now the seventh most common cause of death from noncommunicable diseases (NCDs) worldwide. Contrary to CVD, stroke, and respiratory disease, CKD mortality has been rising. Nowadays, CKD and kidney dysfunction are the third fastest-growing cause of death globally. Importantly, CKD is the only NCD to exhibit a continued rise in age-adjusted mortality [15]. By 2040, CKD is projected to be the fifth highest cause of years of life lost (YLL) globally [16].

Many studies have shown variable CKD prevalence, with significant differences in men and women in most, but not all, cases [17,18,19,20,21]. Differences in reported CKD prevalence could be explained by heterogeneity of study design, by selection bias, by the equation used to estimate the eGFR, or by a variety of CKD definitions used across different studies [22]. This should be considered when interpreting and comparing results on CKD prevalence obtained in different national surveys. The other important point is the fact that the true prevalence is probably underestimated due to the lack of screening programs and early detection of kidney dysfunction, which is related to unawareness of the risk associated with the earlier stages of CKD [23]. This poor health literacy is present not only in the general population and in patients, but also in all health-care workers and health-care authorities [24]. CKD is frequently undetected because of its asymptomatic nature and slow clinical course. According to most reports, only approximatively 10% of patients with CKD were aware of having kidney dysfunction, and the majority were undiagnosed until the very late stages, missing the opportunity for interventions that could prevent disease progression before the kidney injury reached the point of no return [1,25,26]. Finally, a debate on how to differentiate the aging kidney from true CKD in elderly people is ongoing [27,28]. The modification of risk factors and the medical treatment of diabetes, hypertension, and CKD itself can improve renal and CV outcomes and slow or prevent progression to end-stage kidney disease (ESKD) [29,30].

The rising burden of CKD should be seriously appreciated and included in global and national health agendas because it is largely preventable and treatable. However, many countries have underdeveloped nephrology workforces and/or are mainly oriented to the provision of treatment for ESKD but not for the early stages of CKD. In Croatia, most nephrologists focus on patients with ESKD, with an excellent kidney transplantation program and well-documented registries of patients undergoing renal replacement therapy. Unfortunately, interest in individuals at the earlier stages of CKD is disproportionally lower, and there are only a few preventive nephrologists. A surge of new drugs that have proven to be effective in preventing CKD and CVD progression (sodium-glucose transport protein 2 inhibitors, glucagon-like peptide-1 receptor agonists, and finerenone) have increased interest not only from nephrologists but also from cardiologists and diabetologists for CKD starting from the early stages, and it is our hope that the future will be better. Accurate information on both the early and advanced stages of CKD in the general population is important for developing proper strategies for screening, diagnosis, prevention, and treatment. Until now, we have lacked data on the prevalence, characteristics, and awareness of CKD in a representative group of Croatian adults. This is particularly important because Croatia is a high-middle-income country ranked in the group of countries with a high CV risk [31]. To obtain this valuable information, we conducted the EH-UH 2 Study, a nationwide, population-based survey of CKD prevalence and awareness, which incorporated both albuminuria and estimates of eGFR based on the Chronic Kidney Disease Epidemiology Collaboration (CKD-EPI) equation.

## 2. Materials and Methods

### 2.1. Study Design and Recruitment

**Figure 1:**
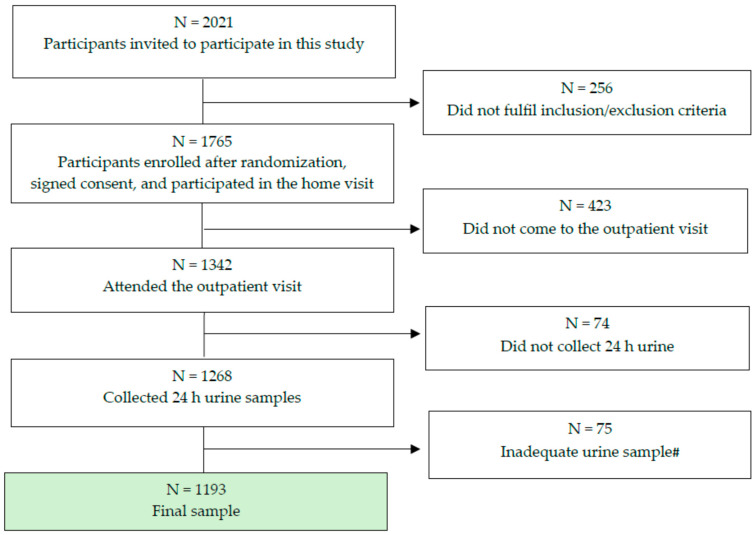
Final number of subjects included in the study. # Either urine volume <500 mL or urine creatinine not in ranges (5.9–26.0 mmol/24 h for men; 4.0–16.4 mmol/24 h women) or urinary tract infection.

**Figure 2:**
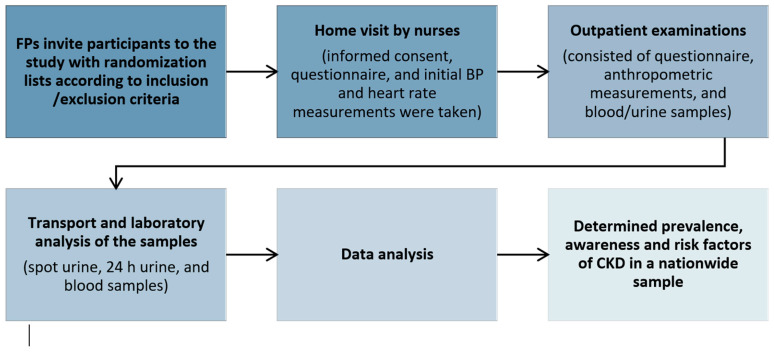
Study design.

This cross-sectional study was part of the EH-UH 2 survey (Epidemiology of arterial hypertension and salt intake in Croatia), a nationally representative survey of non-institutionalized persons in Croatia, which involved collecting anthropometric, demographic, lifestyle, and biological marker data from the general adult population to assess risk factors for cardio–kidney–metabolic health and the prevalence of major NCDs. In this study, we used a stratified random sampling technique, focusing on age and gender, to obtain a representative sample of Croatia’s population, which has approximately 3,871,833 individuals (2021 census). A total of 2021 subjects (18 to 91 years old) were included by random selection from the general population using randomization numbers obtained from the registry of the family physician (FP) (the randomization numbers represented the ordinal numbers of the archive of each family physicians’ practice). The subjects selected via the randomization list were informed about the project by a telephone call from their FP and were included in the study according to the exclusion and inclusion criteria. The exclusion criteria for participation in the project were persons with a terminal illness, dementia, paresis, amputation or immobilization of a limb, acute illness, or convalescing after surgery, pregnant women, lactating mothers, persons with a COVID-19 infection within the last three months, those who were prescribed therapy with diuretics in the last two weeks prior to the urine sample collection date, and unsigned consent to participate in the research, while the inclusion criteria were individuals over 18 years of age who signed their consent to participate in the research. Being on dialysis and after kidney transplantation were not exclusion criteria. However, no subject reported being on any type of renal replacement therapy. In a case when the invited subject had one or more exclusion criteria, the FP contacted the next subject from the randomization list. The final study sample consisted of 1765 participants, representing a sampling rate of 0.0456%, as illustrated in Figure 1. The participation rate (i.e., attending the outpatient visit) was 76%. After the subjects had been included in the study, the nurses, members of the mobile examination team (MET), made an appointment for a home visit. The home visit consisted of obtaining signed consent to participate in the study, giving the subjects their personal identification code, and taking the first measurements of blood pressure (BP) and the heart rate. The subjects were given instructions on how to properly collect and handle urine during a 24 h period and were instructed to fast for 12 h prior to the examination at which the blood samples would be taken. At the end of the home visits, the subjects were invited for outpatient examinations. This examination was carried out in three steps: (a) a questionnaire survey, (b) physical measurements, and (c) having blood drawn, spot urine sample collections, and 24 h urine collections. Nurses, physicians, pharmacists, fellows, residents, and medical students who were members of the MET were educated to collect the study and clinical data in a standardized manner (Figure 2).

### 2.2. Questionnaire

A questionnaire (face-to-face interview) was used to collect data on the participant’s demographics (age, sex, and place of residence), socio-economic status (SES), lifestyle (physical activity, smoking habit, alcohol consumption, diet, frequency of high salt food consumption, fruit and vegetable consumption, and knowledge of dietary salt), personal and family history of cardio–kidney–metabolic diseases, and drug therapy. The subject’s smoking habit was coded as current smoker, former smoker, and never smoked. Pack years were calculated as the number of packs the subject smoked per day multiplied by the number of years the patients had smoked. Heavy alcohol use was defined as consumption of ≥8 drinks/week for women or ≥15 drinks/week for men. SES was measured with two indicators: education and occupation. By answering the question on educational attainment, the subjects were coded as less than primary school (<4 years), primary school (4 to ≤8 years), high school (8–12 years), or university degree (>12 years). Professional qualifications were coded as no college, college, bachelor’s degree, or master’s degree. Physical activity was defined as active (≥75 min/week of vigorous intensity or ≥150 min/week of moderate or an equivalent combination of moderate and vigorous intensity activity), intermediate (5–74 min/week of vigorous intensity or 10–149 min/week of moderate or an equivalent combination of moderate and vigorous intensity activity), or inactive [32]. The poverty–income ratio (PIR) was calculated as family income categorized as ≤130% or >130% based on commonly used thresholds. Each participant’s marital status was described with one of the following categories: married/cohabiting, single, separated/divorced, or widowed.

### 2.3. Anthropometry (Physical Measurements)

The outpatient examinations of the subjects included anthropometric data measurements, measurement of brachial BP, central BP, and arterial stiffness i.e., pulse wave velocity (PWV), recording of an electrocardiogram (ECG), and metabolic scale measurements followed by fasting blood sampling and collecting a morning spot urine sample. Seven days prior to the outpatient examinations, the subjects were reminded to collect and bring a 24 h urine sample from the previous day. Office BP measurements were performed according to the European Society for Hypertension guidelines and the recommendations of the Croatian Society for Hypertension [33,34]. BP was measured on both arms, and if there was a difference in BP between the left and right arm, the higher value was taken as relevant, and the BP was measured on that arm. If there was no difference in BP between the left and right arm, BP was measured on the non-dominant arm. BP and heart rate were measured using an oscilometric device (OMRON M6 Comfort smart cuff). The first measurement was discarded, and an average of the last two readings was used. Hypertension was defined as anyone with systolic BP ≥ 140 and or diastolic BP ≥ 90 mm Hg or taking medication for hypertension. Diabetes was defined as fasting blood glucose > 7 mmol/L and/or taking antidiabetic drugs [35]. The person’s weight and height were measured while they were clothed only in their underwear. A metabolic scale (OMRON BF511) was used for weight measurements to the nearest 100 g, and height was measured with a height rule (stadiometer) rounded to the nearest centimeter. Body mass index (BMI) was calculated by dividing weight in kilograms by height in meters squared (kg/m^2^). Obesity was defined as BMI ≥ 30, overweight as 25 ≤ BMI < 30, and overweight/obesity was defined as BMI ≥ 25 [36]. Waist circumference (WC) was used as a measure of central adiposity (defined as WC > 88 cm for women and >102 cm for men). PWV was calculated from a pulse wave obtained over the brachial artery using an IEM Mobil-O-Graph PWA monitor, and ePWV was calculated using a validated equation (ePWV = 9.587 − (0.402 × age) + [4.560 × 0.001 × (age2)] − [2.621 × 0.00001 × (age2) × systolic AT] + (3.176 × 0.001 × age × systolic AT) − (1.832 × 0.01 × systolic AT)) [37].

### 2.4. Laboratory Analysis

The fasting blood samples and morning urine samples were analyzed using standard laboratory methods. About 8.5 mL in a tube with Silica Clot Activator-SST for the biochemistry tests (BD Diagnostic, Sparks, MD, USA) and 50 mL of the morning urine sample were collected in plastic containers for each subject. Blood samples were centrifuged for 10 min at 3500 rpm within 2 h of collection. All samples were transported the same day to the Department of Laboratory Diagnostics, University Hospital Centre Zagreb, where they were immediately analyzed. Serum and urine creatinine were measured on the Abbott Alinity CC analyzer using the enzymatic method with creatininase traceable to isotope dilution mass spectrometry (IDMS) (Alinity, Abbott, Chicago, IL, USA). Calibration was performed once per lot of reagents using the same company’s calibrators traceable to the IDMS method and the NIST reference material SRM 967 (substance creatinine purity of 99.7 ± 0.3%) for the serum and the NIST reference material SRM 914a (substance creatinine purity of 99.7 ± 0.3%) for the urine samples. Urinary albumin was measured in the 24 h urine sample using the immunonephelometric method on the BN II nephelometer, (Siemens Healthcare Diagnostics Inc., Tarrytown, NY, USA) standardized using primary ERM-DA470 calibrators with a method sensitivity of 3.0 mg/L. Continuous internal quality control was performed throughout the study using quality control materials provided by the respective manufacturers. In the serum samples, additional tests were subsequently performed using standard laboratory methods on the Alinity analyzer (Abbott, Abbott Park, Illinois, IL, USA): triglycerides using photometry with glycerol phosphate oxidase (GPOPAP), total cholesterol using photometry with cholesterol oxidase (CHOD-PAP), HDL cholesterol using the homogeneous enzyme immunoinhibition method, and LDL- cholesterol using the homogeneous enzyme colorimetric method on the same platform, with original reagents from the same manufacturer: Lp (a) using the immunoturbidimetric method on polystyrene particles, NTproBNP and Troponin I hs using the chemiluminescent immunochemical method (CMIA), Apo (A), and Apo (B) using the immunoturbidimetric method on polystyrene particles, uric acid using the photometric method with uricase, glucose using UV photometry with hexokinase, and serum electrolytes using the indirect potentiometric method. Insulin was measured with the Electro Chemi Luminescence Immunoassay (ECLIA) method on the Cobas e 411 instrument (Cobas Roche, California, CA, USA) and tTG IgA using the chemiluminescence method (CLIA) on the BioFlash instrument (Biokit S.A., Barcelona, Spain). The full blood count was determined according to the principle of laser light scattering technology on an Sysmex XN-1000 analyzer (Sysmex Corporation, Kobe, Japan).

The eGFR was calculated for adults aged 18+ years using the Chronic Kidney Disease Epidemiology Collaboration (CKD-EPI) equation based on the serum creatinine values [38]. The eGFR values (mL/min/1.73 m^2^) were grouped into six categories based on the Kidney Disease Improving Global Outcomes (KDIGO) 2012 classification recommendations, as follows: G1: ≥90 mL/min/1.73 m^2^ (normal or high); G2: 60–89 mL/min/1.73 m^2^ (mildly decreased); G3a: 45–59 mL/min/1.73 m^2^ (mildly to moderately reduced); G3b: 30–44 mL/min/1.73 m^2^ (moderately to severely decreased); G4: 15–29 mL/min/1.73 m^2^ (severely decreased)’ and G5: <15 mL/ mL/min/1.73 m^2^ (kidney failure) [29]. The categories of albuminuria based on the urine albumin-to-creatinine ratio (ACR) were classified according to the recommendations of the KDIGO classification into three categories, as follows: A1: <30 mg/g (normal to mildly increased); A2: 30–300 mg/g (moderately increased); and A3: >300 mg/g (severely increased). Increased albuminuria was defined as an ACR ≥ 30 mg/g.

### 2.5. Statistical Analysis

The statistical analysis was performed using SPSS version 23.0 (IBM Corp., New York, NY, USA). The normality of data distribution was tested using the Kolmogorov–Smirnov test. Preliminary analyses were performed to ensure no violation of the assumptions of normality, linearity, and homoscedasticity. The categorical data are expressed as numbers and frequencies. Correlations were obtained using Pearson’s test for normally distributed variables and Spearman’s rank correlation for non-normally distributed variables. The results are reported as the mean (SD and/or 95% Confidence Interval CI), median and interquartile range (IQR) or percentage, as appropriate. Non-normally distributed data are expressed as the median and interquartile range, and the Mann–Whitney U-test was used for comparison between two groups. The categorical variables were compared using the χ^2^-test. The Pearson correlation coefficient was calculated to obtain information about the relationships between the variables. Firstly, the prevalence of different stages of CKD was estimated and expressed as counts and proportions (%). To account for the stratified random sampling method, weighted statistical methods were applied to produce nationally representative CKD prevalence estimates [39]. Secondly, descriptive analyses were performed to present the demographic, socio-economic, behavioral, and cardio-metabolic risk factors of subjects with CKD. Univariate and multivariate logistic regression analyses were used to determine the associations between the parameters and CKD. The results are expressed in terms of the odds ratio (OR) and the respective 95% CI. Finally, multivariable logistic regression analyses were performed to identify the independent contribution of important factors to the risk of having CKD. Based on a literature review and on statistical criteria (variables showing *p* < 0.05 in the univariate analyses), the following variables were introduced in the multivariable models: Model 1: adjusted for age and gender; Model 2: further adjusted for ePWV; Model 3: additionally adjusted for diabetes; Model 4, Model 5, Model 6, and Model 6: further adjusted for urea, uric acid, and serum potassium, respectively. We analyzed the prevalence of subjects who were aware and unaware of having CKD, their characteristics, and their association with risk factors using regression analyses. A two-sided *p*-value < 0.05 was considered statistically significant.

The survey was carried out in accordance with the Declaration of Helsinki and Good Clinical Practice [40]. Ethical approval for the survey was obtained from the Ethics Committee of the School of Medicine, University of Zagreb, and the participants were provided a written informed consent form to sign.

## 3. Results

### 3.1. Overall Prevalence of CKD

The estimated weighted (age-standardized to the Croatian adult population) overall prevalence of CKD (any stage) was 17.1% (95% CI: 8.0–15.0), and the prevalence of CKD ≥ G3A A2 was 9.8% (95% CI: 6.6–9.5). Men had a higher prevalence compared to women (11.8%; 95% CI: 7.6–12.8 vs. 7.9%; 95% CI: 5.2–8.4; *p* < 0.001) (Table 1). The overall crude prevalence of CKD in the population, defined as an eGFR < 60 mL/min/1.73 m^2^, was 9.5% (men vs. women: 12.8% vs. 7.8%; *p* < 0.001). The weighted prevalence of CKD defined by an eGFR <60 mL/min/1.73 m^2^ was lower in the whole group (7.9%; 95% CI: 6.5–9.3; men vs. women: 10.7%; 95% CI: 8.0–13.3 vs. 6.5%; 95% CI: 4.8–8.1; *p* < 0.001).

### 3.2. Prevalence of Albuminuria

The weighted prevalence of albuminuria, using an ACR cut-off of 30 mg/g, was 15.1%, with a significantly higher prevalence observed in men than in women. Table 2 shows the percentages of subjects in the KDIGO categories, including estimates of the crude and weighted prevalence of the subjects with cardio-renal risk based on both the eGFR and ACR. Most subjects were in stages G1 and G2 (weighted prevalence 71%), with 57.7% of them in stages G1A1 plus G2A1, while 11.7% and 0.6% were in stages G1A2 plus G2A2, and G1A3 plus G2A3, respectively.

### 3.3. Estimated CKD Population in Croatia

Based on these results, we estimate that Croatia has approximately 311,441 adults (aged 18–90 years) with CKD stage ≥ G3A A2. Very high, high, and moderate cardio–kidney risk was found in 1.7%, 4.5%, and 16.6% of the adult Croatian population, respectively.

### 3.4. Demographic, Social, Behavioral, and Clinical Characteristics

The differences in demographic, social, behavioral, and clinical characteristics between the CKD and non-CKD populations are shown in Table 3. The CKD group was older, included more men, and had higher systolic blood pressure, with more untreated and uncontrolled hypertension and a higher prevalence of diabetes. Additionally, this group had a greater proportion of overweight and obese individuals with a higher visceral fat circumference and higher arterial stiffness, as estimated by PWV and ePWV. Fewer smokers were observed in the CKD group, but no significant differences in daily salt intake were found. The CKD participants had lower personal and family monthly incomes and were less educated, with fewer professional qualifications.

### 3.5. Cardiovascular and Biochemical Associations with CKD

There were higher rates of ischemic stroke, heart failure, and atrial fibrillation in the CKD group, though differences in hemorrhagic stroke and myocardial infarction did not reach statistical significance. In addition to anticipated differences in kidney biomarkers (serum creatinine, urea, eGFR, and ACR), the CKD participants had higher levels of uric acid, triglycerides, potassium, and cardiac biomarkers (NTproBNP and Troponin I hs), along with total, HDL, and LDL cholesterol.

### 3.6. Factors Associated with CKD

The univariate logistic regression analysis showed that CKD was significantly associated with age, male gender, low family income, systolic blood pressure, obesity (particularly visceral), ePWV, and the presence and duration of hypertension and diabetes. A positive history of ischemic stroke, heart failure, and atrial fibrillation was also associated with CKD. Among the biochemical parameters, fasting blood glucose, uric acid, lipids, NTproBNP, and Troponin I hs were significant predictors of CKD (Table 4). The multivariate regression analysis revealed that male gender (OR 4.2), older age (OR 0.82), ePWV (OR 1.65), diabetes (OR 2.74), urea (OR 0.64), uric acid (OR 0.99), and serum potassium (OR 0.43) were independently associated with higher CKD prevalence (Table 5).

### 3.7. CKD Awareness

CKD awareness in the population was 9.5%. Individuals unaware of their CKD status tended to be older, had lower incomes and qualifications, were less educated, and more frequently had diabetes and poorly controlled hypertension, as well as milder renal impairment (Table 6).

## 4. Discussion

To the best of our knowledge, the EH-UH-2 study is the first study estimating CKD prevalence not only in Croatia, but also in South-East Europe and the Balkan region at the national level based on a large random sample of the adult population according to the KDIGO guidelines using recommended measures andCKD stages defined by the eGFR (CKD-EPI) and ACR. The weighted prevalence of CKD stages 1–5 was 17.1%, which is higher than the 10.6% and 9.1% reported in worldwide analyses [1,41]. The weighted prevalence of CKD stages 3–5 and albuminuria (A2 plus A3) was also higher in our studied population (9.8% and 15.1%, respectively) than in a recent report (5.4% and 5.4%, respectively) [41]. However, our results are much closer to the results of a recent meta-analysis of 100 studies with almost 7 million patients, which reported a global prevalence of 13.4% for CKD stages 1–5 and 10.6% for CKD stages 3–5 [42]. On the contrary, a CKD prevalence of approximatively 6% has been found in Italy, Luxembourg, and Poland [43,44,45]. Our finding of a predominance of early CKD stages is in concordance with all other reports. Direct comparison of various national surveys is very difficult and should be performed very vigilantly. The characteristics of the enrolled population, study design, CKD definitions, and eGFR equations used could be reasons for significant differences among various surveys. Already in 2016, DeNicola and Zoccali pointed out this issue [46]. One of the most important causes for discrepancy in reported results is the usage of different equations. Therefore, our results on CKD prevalence are much more like the results of the studies that used the CKD-EPI equation: 8.7%, 9.1%, 10.0%, 11.5%, 11.9%, and 12.5% in India, Malaysia, Switzerland, Australia, England and Canada, respectively [44,47,48,49,50,51,52].

Using a single cut-off point of an ACR > 30 mg/g, in our cohort, the weighted prevalence of albuminuria was 15.1%, being higher in men than in women. Our results are in concordance with reports from China (19%) and Canada (20%), but higher than results obtained in the US (11.7%), Australia (6%), Italy (4.8%), and Japan (4.6%) [43,53,54,55,56,57]. Observed differences in the albuminuria prevalence could be explained by different definitions and methods, but also strongly depend on the characteristics of examined population. The observed higher prevalence of albuminuria in our study was related to a high prevalence of diabetes, hypertension, and overweight/obesity in the Croatian population. The results from the Health Survey for England showed an increased prevalence of albuminuria with a slight J-shape pattern, where 88% of albuminuria was observed in people with an eGFR > 60, which agrees with our results [58].

In our study, estimates of the prevalence of subjects in specific KDIGO CKD GA categories showed that very few subjects had a high (4.5%) or very high (1.7%) cardio-renal risk considering the combined measure of the eGFR and ACR levels. Most subjects (71.9%) had a normal eGFR (≥60 mL/min/1.73 m^2^) and normal albuminuria (ACR < 30 mg/g), and 14.9% had a normal eGFR but an ACR > 30 mg/g. These results are concordant with reports from other countries [43,44,45,49,53]. Our results on the prevalence of each KDIGO category in our general population are completely in line with the reported international total combined prevalence [59]. We can estimate that in Croatia, more than 311.000 adults between 18 and 90 years of age have CKD stage ≥ G3A A2. A high and a very high risk were estimated to be present in 177.117 subjects, and these subjects should be referred to a nephrologist. However, this would be a very difficult task as Croatia is lacking nephrologists, as in many other countries. Therefore, the Croatian Renal Association and Croatian Hypertension League have started a program of education of family physicians by preparing a curriculum on nephrology and a curriculum on hypertension. Furthermore, we succeeded in negotiating with the national insurance company, which is now fully covering the costs for determination of the ACR in primary, secondary, and tertiary care for all subjects with a high cardio–kidney–metabolic risk. This will hopefully improve earlier detection not only of patients in advanced CKD stages, but also those at the beginning of the cardio–kidney–metabolic continuum who have a moderate risk. According to our estimates, there are 522.065 adult citizen in Croatia in this category, and they deserve particular attention. In the long term, they can obtain the most benefit not only from new cardio-renal protective drugs, but also from changing a poor lifestyle. To increase awareness and to improve the health literacy of the general population, the Croatian Hypertension League has established an educational digital platform named Hunting the Silent Killer, where one of the most important tasks is education on how to change a poor lifestyle (https://tihiubojica.hr/ accessed on 18 August 2024)

Hypertension, diabetes, and overweight/obesity were significantly more prevalent in our CKD group compared to the subjects not having CKD. There were significantly fewer controlled and many more untreated hypertensive patients in the CKD group, which could contribute to higher CV risk and more CV morbidity in these patients. A history of atrial fibrillation, heart failure, and ischemic stroke, were significantly more frequently presented in the CKD group than in the non-CKD group. Myocardial infarction and hemorrhagic stroke were also more prevalent in the CKD group than in the non-CKD group, but the difference was not significant. An association between CKD and CV and cerebrovascular morbidity has also been reported in other studies, but wide variability in its prevalence was found, which also could explain observed differences in CKD prevalence among various surveys [60]. In the univariate logistic regression, we found various demographic, socio-economic, behavioral, and health-related risk factors to be associated with CKD. In the multivariate regression analysis, older age, male gender, diabetes, uric acid, and ePWV were independently associated with higher CKD prevalence. Similar results have been reported by other authors, with slight differences depending on the basal characteristics of the enrolled populations. In the GBD study, impaired fasting plasma glucose, high BP, a high BMI, and a diet high in sodium were risk factors for CKD, but high BP contributed mostly to a CKD burden in east Asia, eastern Europe, tropical Latin America, and western sub-Saharan Africa, whereas high fasting plasma glucose was the leading risk factor for CKD in all other regions [1]. Even in non-obese individuals, a clustering of CV risk factors has an impact on CKD. In a Japanese study, adult non-obese subjects with at least three metabolic factors had an equal or slightly higher risk of renal dysfunction than obese subjects with ≥3 metabolic factors [61]. In our group, uric acid was significantly associated with CKD prevalence. The association of uric acid with CKD and hyperuricemia with progression of disease remains controversial and debatable. A systematic review of 23 studies containing 212,740 subjects found a pooled prevalence of 43.6% hyperuricemia in patients with CKD globally, and it was reported as 67.4% and 32.6% in the cases of male and female patients, respectively [62]. In our population, we failed to find differences in salt consumption between the CKD and non-CKD populations. Some other authors found the sodium-to-potassium ratio to be a more important risk factor for CKD than solely salt ingestion [63]. Our results on this topic will be published soon.

A low family monthly income and less education were associated with CKD prevalence in our cohort. These results are in line with reports that a significant increase in CKD DALYs globally was most pronounced in the middle and low-middle SDI quintiles [1,5]. One study reported important differences by geographic region classified by income level that had an impact on CKD age-standardized prevalence in both men and women: 8.6% and 9.6%, respectively, in high-income countries and 10.6% and 12.5%, respectively, in low- and middle-income countries [59]. In the multicenter German Chronic Kidney Disease cohort, the subjects with low educational attainment (vs. high) had a higher risk of mortality and kidney failure, particularly for diabetic kidney disease [64]. Although not unanimously reported, in many studies, the prevalence of CKD tends to be higher in women than in men [65]. One important explanation for such finding is that a single cut-off of <60 mL/min per 1.73 m^2^ for the CKD definition may result in over-diagnosing CKD in women [65]. The results on a higher prevalence in women reported in many studies are in contrast with observations from in vitro experiments, which found a protective effect of estrogen and a detrimental effect of testosterone on non-diabetic CKD [21]. In addition to the hormonal reasons, differences in lifestyle and socio-economic factors were reported to be associated with such findings [66]. These results conflict with data that indicate a higher prevalence and incidence of CKD, faster progression, and a higher mortality rate in men [19,41,65,66,67,68]. Overall, the decline of eGFR with age in men has been reported to be 0.92 mL/min per 1.73 m^2^ compared with 0.75 mL/min per 1.73 m^2^ in women [69]. In our study, like in Italian and Spanish cohorts, the prevalence of CKD was higher in men than in women [43,70]. We failed to find differences in age or prevalence of hypertension between the men and the women, but the men were significantly more overweight/obese and more frequently had diabetes, ingested more salt, and had higher values of BP, fasting blood glucose, uric acid, and faster ePWV. All these characteristics made them more prone to CKD than the women.

Only 9.5% of our CKD patients were aware of having CKD. This is in line with other reports that only 6% of the general population and 10% of the high-risk population are aware of their CKD [25,43,45]. In our group, those subjects unaware of having CKD were older with lower income and qualifications, less educated, and more frequently had diabetes, hypertension (more uncontrolled), and milder renal impairment. The GBD Study has shown that CKD awareness is generally low worldwide, with significant disparities between high-income and low-income countries [1]. The observed association of unawareness of CKD with lower education and a poorer socio-economic index is in line with the general observation that CKD is particularly detrimental in vulnerable subpopulations. Patients with milder renal impairment were also less aware, and our results are like the results of other studies [25,26,43,45,71]. This could be explained by having fewer (or no) symptoms of CKD in the early stages compared to more advanced CKD stages. Interestingly, in our group awareness was associated with the male gender like it was reported in Poland [45]. A higher prevalence of hypertension, diabetes, atrial fibrillation, and, particularly, a lower percentage of controlled hypertensive patients in the unaware group is particularly disturbing and disappointing. This reflects not only poor awareness of patients or subjects with CKD, but is also an indicator of clinical inertia and poor health literacy of physicians. Namely, most of them, at least the treated hypertensive patients, were under the control of physicians who obviously neither checked kidney function nor informed their patient that they had CKD. The International Society of Nephrology’s “Global Kidney Health Atlas” reports that many countries lack adequate CKD awareness programs, especially in regions with limited health-care access, underscoring the critical need for enhanced public health initiatives to improve CKD awareness, early detection, and management globally. Increased efforts in education, screening, and health-care access are essential [72]. Our results confirm that this is an utmost need for Croatia, where both poor awareness and clinical inertia should be improved.

### Strengths and Limitations

This study has several important strengths. Our results are based on a large nationwide, population-based sample of general adults in Croatia. We weighted data, which allowed us to estimate a population-representative prevalence of CKD. Kidney dysfunction was determined following the most recent guidelines, combining the CKD-EPI, eGFR, and ACR, enabling us to define the CKD GA stages. One of the important strong points is the fact that we determined the ACR from adequate and reliable 24 h urine samples. Furthermore, we analyzed a broad set of risk factors for CKD, including demographic, socio-economic, clinical, and lifestyle variables. An obvious limitation is the cross-sectional design of the study. Secondly, we measured the eGFR and ACR only once, which could overestimate the prevalence. The next limitation is that we excluded subjects in residential care potentially having CKD, which could underestimate the prevalence. Another important limitation was the lack of data on cystatin C-based eGFR, which would increase the accuracy. And finally, we did not consider aging, did not use different suggested cut-offs for different age groups, and did not use the BIS equation for elderly people. The definition of an eGFR < 60 mL/min per 1.73 m^2^ may not be optimal for defining CKD in elderly people, because 50% of persons older than 70 years might be labeled as having CKD, while it is more likely that they have only physiologic aging of the kidneys [73]. In recent years, the need for an age-dependent definition of CKD has been proposed, and some authors have suggested that the eGFR thresholds for defining CKD could be adapted to <75 m/min per 1.73 m^2^ in youth, <60 mL/min per 1.73 m^2^ for middle-aged adults, and <45 mL/min per 1.73 m^2^ for older adults [27,28]. Using various definitions and/or different equations for elderly people could bring our results closer to the real prevalence. However, it would not enable us to compare our results with worldwide published results on CKD prevalence because most studies have used a single cut-off for CKD, and in the last decade, the majority have used the CKD-EPI equation and KDIGO staging. Furthermore, large population-based studies indicate that even in older adults at lower risk of kidney failure, stage 3 CKD is associated with an elevated risk of mortality, CV events, and acute kidney injury [74]. Therefore, KDIGO guidelines support the use of an eGFR threshold of <60 mL/min/1.73 m^2^ to define CKD, regardless of age group.

## 5. Conclusions

This is the first evidence-based report on the prevalence, characteristics, and awareness of CKD not only in Croatia but also in this part of Europe. A high prevalence of CKD and an unfavorable CV risk profile associated with very poor awareness strongly point to the need for systematic actions in Croatia, which will ameliorate the high burden of this important public health problem. The nephrology community, together with hypertensiologists, diabetologists, and cardiologists, and with the strong support of national health authorities, should jointly organize continuous activities that will increase awareness and improve clinical inertia and help in earlier detection, organization of primordial and primary prevention, and evidence-based medical treatment, also considering knowledge of epidemiology and pathophysiology. Some of the first steps have already been taken: all physicians in Croatia can determine the ACR in all subjects with a high cardio–kidney–metabolic risk, and this is fully covered by the national insurance company. Additionally, we have started with educating the general population (https://tihiubojica.hr/ accessed on 18 August 2024; https://www.70-26.hr/ accessed on 18 August 2024; https://www.kolesterol.hr/ accessed on 18 August 2024) and health-care workers (https://www.healthmed.hr/ accessed on 18 August 2024; https://hdh.healthmed.hr/curriculum-hypertensiologiae/ accessed on 18 August 2024) [75]. With these actions, we hope to slow the progression of CKD and substantially decrease CV risk. There is no try. Failure is not an option.

## Figures and Tables

**Table 1:** Weighted prevalence of chronic kidney disease in the adult population using various definitions.

	Overall CKD (Any KDIGO Stage)		CKD ≥Stage 3GA A2		CKD <60 mL/min/1.73 m^2^		ACR >30 mg/g	
	%	95% CI	%	95% CI	%	95% CI	%	95% CI
All	17.1	8.0–15.0	9.8	6.6–9.5	7.9	6.5–9.5	15.1	7.0–15.2
Men	19.3	8.3–17.3	11.8	7.6–12.8	10.7	8.0–13.3	17.5	6.9–16.0
Women	14.9	7.1–11.5	7.9	5.2–8.4	6.5	4.8- 8.1	13.5	6.5–10.7

CKD, chronic kidney disease; ACR, albumin-creatinine-ratio; KDIGO, Kidney Disease: Improving Global Outcomes; G, GFR category; A Albuminuria category; CI, Confidence Interval.

**Table 2:** Distribution of adult population based on the eGFR and ACR with prevalence estimates according to KDIGO chronic kidney disease risk groups.

				Albuminuria Categories			
				A1	A2	A3	
				ACR < 30 mg/g	ACR 30–299 mg/g	ACR ˃300 mg/g	Total
eGFR categories (mL/min/1.73 m^2^)	G1	≥90	crude weighted 95% CI	55.4 **45.4** 43.1–47.8	9.8 **8.1** 6.7–9.6	0.2 **0.2** 0.002–0.4	65.4 53.7
	G2	60–89	crude weighted 95% CI	16.9 **13.3** 11.6–15.0	4.3 **3.6** 2.6–4.5	0.5 **0.4** 0.1–0.8	21.7 17.3
	G3A	45–59	crude weighted 95% CI	5.9 **4.9** 3.8–6.0	2.3 **1.9** 1.2–2.7	0.4 **0.3** 0.04–0.6	8.6 7.1
	G3B	30–44	crude weighted 95% CI	3.5 **2.0** 0.6–5.0	0.2 **0.2** 0.02–0.4	0	3.7 2.2
	G4	15–29	crude weighted 95% CI	0.1 **0.1** 0,05–0.3	0.2 **0.2** 0.02–0.4	0.07 **0.06** 0.006–0.2	0.38 0.36
	G5	<15	crude weighted 95% CI	0.07 **0.06** 0.002–0.02	0.07 **0.06** 0.006–0.12	0.08 **0.07** 0.006–0.2	0.22 0.19
			crude weighted	81.87 68.37	16.88 14.07	1.25 1.04	100
CKD risk			%		estimated number		
			crude	weighted	of Croatian adult population		
Low risk			72.3	58.7	1846.099		
Moderate risk			20.0	16.6	522.065		
High risk			6.5	4.5	141.523		
Very high risk			1.1	1.1	35.594		
CKD ≥ G3A A1			12.8	9.8	311.441		

A, Albuminuria category; ACR, albumin–creatinine-ratio; G, GFR category; CKD, chronic kidney disease; CI, Confidence Interval. Background Color Key: Green—no other signs of renal damage, low risk of ESKD; Yellow—medium risk of ESKD; Orange—high risk of ESKD; Red—very high risk of ESKD.

**Table 3:** Characteristics of subjects with chronic kidney disease and subjects with normal kidney function.

	CKD Population (N = 117)		Non-CKD Population (N = 1076)		χ²	*p*
	Mean (SD)	95% CI	Mean (SD)	95% CI		
	Median (IQR) #		Median (IQR) #			
Age #	72 (65–75)	68.3–72.3	58 (46–75)	55.34–56.9		<0.001
Gender (men) %	49.6	40.7–58.5	33.8	31.1–36.6	57.42	<0.001
Hypertension duration (years) #	10 (5–18)	10.7–16.2	9.0 (4.0–14.0)	9.9–11.5		0.005
Systolic BP (mmHg) #	137 (125–151)	134.8–144.4	131 (120–144)	143.0–134.1		0.001
Diastolic BP (mmHg) %	79.7 (11.3)	77.3–82.1	82.6 (10.3)	82.2–83.2		0.011
Hypertension (yes) %	72.1	63.5–79.6	61.6	58.6–64.2		NS
Treated controlled (yes) %	20.7	15.7–30.9	23.4	21.0–25.9		
Untreated (yes) %	28.9	21.2–37.6	20.9	18.6–23.4	7.57	0.057
Heart rate (bpm) #	75.3 (16–83.6)	74.2–80.4	75.0 (67–83)	74.8–76.1		0.168
Height (cm) #	166 (160–175)	169.1–173.5	168 (162.0–175)	168.3–169.4		0.198
Weight (kg) #	82 (63–93)	81.6–88.8	80 (69.0–91.4)	80.4–82.4		0.191
Body mass index (kg/m^2^) #	29.3 (26.2–32.5)	27.9–29.9	27.9 (24.7–31.3)	28.1–28.7		0.008
BMI category (kg/m^2^) %						
25–29.9	35.3	26.8–44.6	39.0	36.1–41.9		
30.0–34.9	36.1	27.5–45.4	23.1	20.6–25.6	14.52	0.013
35.0–39.9	9.2	4.7–15.9	7.3	5.9–9.0		
Waist circumference (cm) #	103.5 (95.0–110.2)	100.5–105.2	97.0(87.0–108.0)	96.7–98.5		<0.001
WC pathologic #	80.5	72.2–87.2	64.9	62.0–67.7	11.69	0.003
Body surface area (m^2^) #	1.95 (1.180–1.95)	1.95–2.05	1.93 (1.77–2.09)	1.93–1.96		0.426
Smokers (yes) %	10.9	6.1–17.5	26.4	23.9–29.0	15.04	<0.001
Daily salt intake (g/day) #	7.2 (5.0–12.4)	8.1–10.3	8.4 (5.6–11.4)	8.5–9.0		0.234
Daily salt intake > 5 g %	24.4	17.1–33.0	19.0	16.8–21.4		NS
ePWV (m/s) #	12.5 (10.6–13.7)	11.4–12.3	9.5 (7.9–11.2)	9.5–9.8		<0.001
Monthly income (<300 Eu) %	42.6	34.0–51.6	30.2	27.6–32.9	14.45	0.006
Family monthly income (<300 Eu) %	22.5	15.6–30.7	8.4	6.9–10.2	34.8	0.001
Education (years) %						
No school	0.8	1.1–6.0	0.8	0.4–1.5		
<4	3.1	0.7–9.4	1.2	0.7–2.0		
4–8	27.9	10.5–27.3	15.6	14.7–19.0	16.44	0.002
8–12	45.1	41.4–62.9	55.4	51.7–57.4		
≥12	21.7	16.9–35.8	26.9	24.1–29.1		
Professional qualification %						
No college	38.0	19.1–39.3	21.1	20.1–24.8		
College	38.8	34.0–55.3	42.7	39.3–45.0	21.043	<0.001
Bachelor’s degree	12.4	7.9–23.4	17.1	14.8–18.9		
Master’s degree	9.3	5.5–19.5	17.3	14.9–19.2		
Stroke ischemic %	4.7	1.7–9.8	2.4	1.6–3.4	6.358	0.042
Stroke hemorrhagic %	0.8	0.0–4.2	0.3	0.1–0.9		NS
Myocardial infarction %	3.1	1.3–7.7	2.5	1.7–3.6		NS
Heart failure %	3.9	1.3–8.8	0.8	0.3–1.4	13.06	0.001
Atrial fibrillation %	10.1	5.5–16.6	2.8	1.9–3.9	10.25	0.006
Fasting blood glucose (mmol/L)	5.4 (4.7–6.7)	5.6–6.6	4.9 (4.4–5.2)	5.0–5.2		<0.001
Diabetes %	29.5	21.8–38.1	14.0	12.1–16.1	36.40	<0.001
Urea (mmol/L) #	7.5 (6.2–9.2)	7.6–9.3	5.2 (4.4–6.2)	5.3–5.5		<0.001
Serum creatinine (µmol/min) #	110 (92.0–120.5)	119.6–138.7	68.0 (61.0–79.0)	69.5–71.0		<0.001
eGFR (mL/min/1.73 m^2^) #	54.7 (44.3–58.0)	45.8–50.6	92.2 (81.7–101.4)	90.0–91.8		<0.001
Uric acid (µmol/L) #	360 (304–422.1)	368.2–411.7	280 (234–338)	285.8–294.4		<0.001
Total cholesterol (mmol/L) #	4.9 (4.1–5.9)	4.7–5.2	5.3 (1.1)	5.3–5.4		0.003
Triglycerides (mmol/L) #	1.5 (1.1–2.1)	1.5–2.2	1.3 (0.9–1.9)	1.5–1.6		0.009
LDL cholesterol (mmol/L)#	2.8 (2.1–3.5)	2.6–3.0	3.1 (2.4–3.9)	3.1–3.2		<0.001
HDL cholesterol (mmol/L) #	1.3 (1.1–1.5)	1.2–1.4	1.4 (1.1–1.6)	1.4–1.5		0.022
Serum potassium (mmol/L) #	4.7 (4.3–5.6)	4.6–4.9	4.5 (4.3–4.8)	4.5–4.6		0.001
NT pro BNP (mmol/L) #	152.2 (76–358)	324.0–781.1	73 (41.0–127.2)	109.8–134.1		<0.001
Hs Troponin I (mmol/L) #	5.0 (5.0–5.0)	5.74–13.7	5.0 (5.0–5.0)	5.7–6.5		<0.001
ACR (mg/g) #	17.3 (5.6–44.1)	26.7–291.19	9.4 (4.6–20.8)	22.6–32.2		<0.001
ACR category (mg/g) %						
30–299	25.0	17.3–34.1	16.4	14.2–18.8		
<300	7.1	3.1–13.6	1.0	0.5–1.8	31.37	<0.001

CKD, chronic kidney disease; SD, Standard Deviation; χ^2^, Chi-Square; *p*, *p*-value; CI, Confidence Interval; IQR, interquartile range; BP, blood pressure; BMI, body mass index; WC, waist circumference; ePWV, estimated Pulse Wave Velocity; eGFR, estimated glomerular filtration rate; LDL, Low-Density Lipoprotein; HDL, High-Density Lipoprotein; NT pro BNP, N-terminal pro-B-type Natriuretic Peptide; ACR, albumin-to-creatinine ratio. “%” indicates percentage; “#” indicates Median (IQR).

**Table 4:** Demographic, socio-economic, behavioral, and health-related factors associated with chronic kidney disease in univariate logistic regression.

	Coef (B)	S.E.	Odds Ratio Exp (B)	95% CI Lower Upper		*p*
Age (years)	−1.00	0.012	0.90	0.88	0.92	<0.001
Gender (men)	1.680	0.243	5.36	3.33	8.63	<0.001
Family income < 300 Eu	−1.03	0.31	0.35	0.66	1.80	0.004
Systolic BP (mmHg)	0.810	0.339	2.23	1.15	4.36	0.017
Hypertension (no)	0.481	0.205	1.61	1.08	2.42	0.019
Hypertension duration (years)	−0.026	0.011	0.97	0.95	0.99	0.023
Stroke ischemic (yes)	1.114	0.464	3.04	1.27	7.56	0.016
Heart failure (yes)	1.757	0.603	5.79	1.77	18.87	0.004
Atrial fibrillation (yes)	1.158	0.407	3.18	1.43	7.07	0.004
Body mass index (kg/m^2^)	−0.035	0.018	0.96	0.93	0.99	0.045
Waist circumference (cm)	−0.021	0.006	0.97	0.96	0.99	<0.001
PWV (m/s)	−0.362	0.141	0.65	0.52	0.91	0.01
ePWV (m/s)	−0.431	0.053	0.65	0.58	0.72	<0.001
Fasting blood glucose (mmol/L)	−0.197	0.044	0.82	0.75	0.89	<0.001
Diabetes (yes)	1.32	0.233	3.76	2.38	5.93	<0.001
Uric acid umol/L)	−0.012	0.001	0.98	0.98	0.99	<0.001
Serum creatinine (µmol/L)	−0.372	0.051	0.68	0.62	0.76	<0.001
Urea (mmol/L)	−0.248	0.047	0.79	0.72	0.86	<0.001
Total cholesterol (mmol/L)	0.258	0.085	1.29	1.09	1.53	0.002
HDL cholesterol (mmol/L)	0.818	0.312	2.26	1.22	4.17	0.009
LDL cholesterol (mmol/L)	0.373	0.114	1.45	1.16	1.81	0.001
Triglycerides (mmol/L)	−0.017	0.059	0.89	0.79	0.99	0.046
Potassium (mmol/L)	−0.727	0.189	0.48	0.33	0.81	<0.001
NT pro BNP (mmol/L)	−0.002	0.001	0.99	0.99	0.99	<0.001
hs Troponin I (mmol/L)	−0.004	0.01	0.95	0.93	0.98	<0.001
ACR	−1.0640	0.201	0.34	0.23	0.51	<0.001
ACR 30–299 (mg/g)	−0.745	0.273	0.47	0.27	0.81	0.006
ACR > 300 (mg/g)	−2.742	0.507	0.06	0.02	0.17	<0.001

Coef (B), Coefficient B; S.E., Standard Error; Exp (B), Exponentiated B; CI, Confidence Interval; *p*, *p*-value; BP, blood pressure; PWV, Pulse Wave Velocity; ePWV, estimated Pulse Wave Velocity; HDL, High-Density Lipoprotein; LDL, Low-Density Lipoprotein; NT pro BNP, N-terminal pro-B-type Natriuretic Peptide; ACR, Albumin-to-Creatinine Ratio.

**Table 5:** Multivariate logistic regression analysis estimating independent correlates of chronic kidney disease.

Model	Coef	S.E.	Odds Ratio	95% CI		*p*	Nagelkerke R2	*p* for Change
	(B)		Exp (B)	Lower	Upper			
Model 1							0.287	<0.001
Constant	8.554	0.863	5186.45			<0.001		
Gender	1.780	0.258	5.93	3.58	9.82	<0.001		
Age	−0.106	0.013	0.90	0.87	0.92	<0.001		
Model 2							0.304	<0.001
Constant	8.997	0.936	8075.6			<0.001		
Gender	1.837	0.262	6.280	3.760	10.48	<0.001		
Age	−0.174	0.029	0.841	0.794	0.89	<0.001		
ePWV	0.359	0.138	1.432	1.092	1.878	0.009		
Model 3							0.331	0.557
Constant	8.407	1.108	4479.2			<0.001		
Gender	1.778	0.265	5.917	3.517	9.954	<0.001		
Age	−0.181	0.030	0.834	0.787	0.885	<0.001		
ePWV	0.409	0.141	1.505	1.141	1.984	0.004		
Diabetes	0.995	0.324	2.705	1.144	5.105	<0.001		
Model 4							0.454	<0.001
Constant	10.198	1.096	26,860.47			<0.001		
Gender	1.921	0.299	6.828	3.8	12.2	<0.001		
Age	−0.158	0.032	0.854	0.80	0.91	<0.001		
Diabetes	0.948	0.290	2.579	1.46	4.55	0.001		
ePWV	0.408	0.155	1.504	1.11	2.03	0.008		
urea	−0.503	0.071	0.589	0.51	0.67	<0.001		
Model 5							0.496	0.001
Constant	13.057	1.358	468,266.30					
Gender	1.581	0.315	4.862	2.64	9.00	<0.001		
Age	−0.177	0.034	0.838	0.78	0.89	<0.001		
Diabetes	1.009	0.303	2.743	1.51	4.96	<0.001		
ePWV	0.501	0.161	1.650	1.20	2.26	0.002		
urea	−0.442	0.075	0.643	0.55	0.74	<0.001		
Uric acid	−0.009	0.002	0.991	0.98	0.99	<0.001		
Model 6							0.518	0.001
Constant	17.752	2.091	51,240,062.3			<0.001		
Gender	1.445	0.323	4.240	2.250	7.991	<0.001		
Age	−0.187	0.036	0.829	0.773	0.890	<0.001		
Diabetes	1.005	0.315	2.731	1.472	5.065	0.001		
ePWV	0.552	0.171	1.73	1.242	2.431	0.001		
urea	−0.429	0.80	0.651	0.557	0.762	<0.001		
Uric acid	−0.11	0.002	0.989	0.985	0.993	<0.001		
potassium	−0.837	0.270	0.433	0.255	0.735	0.002		

Coef (B), Coefficient B; S.E., Standard Error; Exp (B), Exponentiated B; CI, Confidence Interval; *p*, *p*-value; ePWV, estimated Pulse Wave Velocity.

**Table 6:** Characteristics of subjects divided into groups depending on CKD awareness.

	CKD Aware N = 11		CKD Unaware N = 106		χ²	p
	Median (IQR)	95% CI	Median (IQR)	95% CI		
Men %	54.5	23.4–83.8	50.0	40.1–59.9	12,653	0.002
Age (years)	71 (62–75)	62.51–75.68	72 (65–79)	69.2–72.9		NS
Smokers %	18.2	27.3–51.8	11.3	6.0–18.9	12,004	<0.017
Hypertension duration (years)	15.5 (5.5–13.2)	54.5–56.1	10.0 (5.0–16.0)	10.2–14.7		NS
Systolic BP (mmHg)	138 (126–138)	129.3–162.6	137.5 (124–153)	135.5–144.2		NS
Waist circumference %	88.9	51.8–99.7	78.4	68.8–86.1	10,222	0.037
Personal income < 300 Eu %	44.3	37.4 -54.3	32.4	1.7–35.2	18,169	0.029
Family income < 300 Eu %	22.6	15.1–31.8	10.1	8.4–12.6	39,534	<0.001
Education < 12 years %	50.0	40.1–55.9	54.8	51.8–57.7	20,269	0.009
No college %	36.8	27.6–46.7	23.5	21.1–26.6	21,771	0.005
Atrial fibrillation %	1.8	0.2–5.1	8.5	4.0–15.5	19,446	0.001
Hypertension %	63.6	30.8–89.1	72.6	63.1–80.9	5583	0.061
Controlled hypertensive %	23.4	21.0–26.9	21.9	14.4–31.0	12,851	0.045
Diabetes %	27.3	17.6–61.0	29.2	20.8–38.9	18,913	<0.001
ePWV (m/s)	11.5 (10.1–13.9)	10.4–13.4	12.0 (10.6–13.4)	11.6–12.4		NS
Urea (mmol/L)	8.2 (3.6)	5.7–10.6	8.0 (3.0)	7.4–8.6		NS
Serum creatinine (µmol/L)	119 (106–132)	101.5–157.0	110 (91.7–118)	106.7–121.6		NS
eGFR (mL/min/1.73 m^2^)	45.2 (38.5–58.0)	38.4–53.6	55.0 (44.5–58.1)	49.5–53.3		NS
Uric acid (µmol/L)	350 (284–475)	287.6–482.9	360.0 (304–417)	350.5–386.4		NS

CKD, chronic kidney disease; χ^2^, Chi-Square; *p*, *p*-value; IQR, interquartile range; CI, Confidence Interval; NS, not significant; BP, blood pressure; ePWV, estimated Pulse Wave Velocity; eGFR, estimated glomerular filtration rate.

## Data Availability

The original contributions presented in the study are included in the article, further inquiries can be directed to the corresponding authors.
